# GOALIATH: a theory of goal-directed behavior

**DOI:** 10.1007/s00426-021-01563-w

**Published:** 2021-07-29

**Authors:** Bernhard Hommel

**Affiliations:** 1 Cognitive Neurophysiology, Department of Child and Adolescent Psychiatry, Faculty of Medicine, TU Dresden, Germany, Schubertstr. 42, 01307 Dresden, Germany; 2 Institute for Psychological Research, Leiden University, Leiden, The Netherlands; 3 Department of Psychology, Shandong Normal University, Jinan, China

## Abstract

Commonsense and theorizing about action control agree in assuming that human behavior is (mainly) driven by goals, but no mechanistic theory of what goals are, where they come from, and how they impact action selection is available. Here I develop such a theory that is based on the assumption that GOALs guide Intentional Actions THrough criteria (GOALIATH). The theory is intended to be minimalist and parsimonious with respect to its assumptions, as transparent and mechanistic as possible, and it is based on representational assumptions provided by the Theory of Event Coding (TEC). It holds that goal-directed behavior is guided by selection criteria that activate and create competition between event files that contain action-effect codes matching one or more of the criteria—a competition that eventually settles into a solution favoring the best-matching event file. The criteria are associated with various sources, including biological drives, acquired needs (e.g., of achievement, power, or affiliation), and short-term, sometimes arbitrary, instructed aims. Action selection is, thus, a compromise that tries to satisfy various criteria related to different driving forces, which are also likely to vary in strength over time. Hence, what looks like goal-directed action emerges from, and represents an attempt to satisfy multiple constraints with different origins, purposes, operational characteristics, and timescales—which among other things does not guarantee a high degree of coherence or rationality of the eventual outcome. GOALIATH calls for a radical break with conventional theorizing about the control of goal-directed behavior, as it among other things questions existing cognitive-control theories and dual-route models of action control.

## Introduction

Goals are shaping our lives: our own goals organize our everyday activities and our long-term lifestyle, and those of others determine the constraints within which the striving for our own goals can unfold. Self-help books and smart software tools support our goal-striving and encourage us to get a better understanding of our goals, and to make them explicit to ourselves and others. Evaluations of job performance and personnel selection are based on the formation and description of personal goals that one wants to achieve. And personal relationships can break apart because of differing goals and goal orientations.

The concept of goals is not just popular in everyday communication, but it also has shaped psychological theorizing. It is true that the relevance of the concept has seen some ups and downs during the development of academic psychology since 1870. Early theorists considered it obvious to consider an important role of goals in understanding human cognition and performance. For instance, proponents of ideomotor theorizing, like Lotze (1852), Harless (1861), and James (1890), tried to develop a mechanistic understanding of how humans translate representations of intended future states (i.e., goal representations) into body movements that are suited to make these states more likely (i.e., goal-striving, goal-directed action). These ambitions were shared by the pioneers of will psychology, like Ach (1910, 1935) and Lewin (1922a, 1922b), even though they were more interested in the motivational power of goals than in their actual mechanics.

However, the increasing dominance of mainly Russian and North American stimulus–response approaches actively suppressed theorizing about goals, up to the point of openly ridiculing theorizing about goal-driven action (Thorndike, 1913). Even though the explicit depreciation of in particular ideomotor theorizing (which was dismissed as merely putting a hyphen between the terms ideo and motor) was shared by Miller et al. (1960), it were these authors (and, arguably, Powers et al., 1960; see Austin & Vancouver, 1996) who put the goal concept back on the main agenda of psychological thinking. Since then, the concept has flourished and pops up in all departments of psychology and the wider areas of the cognitive sciences and neurosciences, and various approaches have emphasized the functionality of goals and all the interesting things that goals can do for us, by constraining action selection and shaping action control (e.g., Miller et al., 1960), steering attention towards action-relevant information (e.g., van der Laan et al., 2017), modulating values and evaluation (see Eccles & Wigfield, 2002), and much more.

Interestingly, however, while the goal concept now enjoys such a widespread popularity, its actual mechanics are still poorly understood. For instance, motivational accounts commonly ask participants what their goal is and test whether and under which circumstances they do perform actions implied by this goal (e.g., Gollwitzer, 1990; Locke et al., 1988)—without even trying to explain what codes, constructs, or networks the verbal report refers to and exactly how these codes, constructs, or networks generate the observed behavior. Even computational models with more mechanistic ambitions commonly reduce goals to not further explained instructions (e.g., Logan & Gordon, 2001) or switches (e.g., Cooper & Shallice, 2000) that somehow “know” when and how to activate hardcoded (i.e., also not further analyzed or explained) task-sets/schemata that somehow bring about the action of interest. These approaches hardly go beyond what we know already: *that* goals can control our action, but fail to explain the *how*. It is this *how* that I shall focus on in the following: How can goals control our action (the processes involved) and what do they consist of (the representations involved) so to achieve that? Accounting for both processes and the representations they operate on lies at the core of mechanistic theorizing. Unfortunately, theorizing about human goals is often not overly mechanistic but, as I shall explain in the next section, descriptive and circular. Then, I shall ask whether the concept of a goal is actually needed and what it needs to account for, ask how a minimal representation of a goal might look, how many goals we pursue at the same time, how we select goals, where goals come from, and what it might mean to have no goals. I shall conclude by pointing out some further implications and drawing some conclusions.

## Meta-theoretical considerations

Theorizing in psychology and the cognitive sciences, including the cognitive neurosciences, often reflects its roots in philosophy and the humanities (see Hommel, 2020). Setting research agendas often begins with taking a particular pre-scientific concept, such as attention, and then starting to explain what it actually is and how it works. The next step commonly consists in recognizing that the meaning of the word in everyday language is hopelessly context dependent and hard to grasp in terms of experimental investigations, and in attempting to better define the concept—without ever questioning the underlying (pre-scientific) assumption that the concept refers to a meaningful subdivision of the human psyche (see Danziger, 1997). The outcome is typically some kind of analytical subdivision, such as the distinction between endogenous and exogenous attention, attention to object and to location, attention to features and to objects, object selection and action selection, attentional focusing and attentional search, and so forth and so on. These different sorts of attention are then studied by often non-overlapping research communities that use different experimental paradigms and publish in different journals. This eventually leads to a multitude of theoretical models and theories that are difficult to relate, even though sometimes some degree of integration will be tried. The kind of theorizing commonly consists in inventing internal systems or neural networks that serve no other purpose than generating the phenomenon under investigation (Hommel et al., 2019; Hommel, 2019a, 2020). The resulting theories are, thus, commonly circular and relatively void of mechanistic considerations: the phenomenon is assumed to be “explained” by having a system or network producing it, so that for instance dual-tasking costs are “explained” by the mere having of a “capacity-limited system” (Hommel, 2020). This is where explanation typically stops or reaches an informational asymptote, which leaves the field with numerous systems and networks. Other fields follow similar routes, also leaving them with numerous systems and networks.

Attempts to reduce this inflated number of mechanisms are hampered by the false belief that the very fact that everyday language has invented a word for a category of phenomena can be taken as sufficient evidence that the mechanisms assumed to underlie these phenomena must show some coherence and create some sort of category as well (Danziger, 1997): attentional mechanisms are assumed to be dedicated to attention, cognitive mechanisms to cognition, affective mechanisms to affect, and so on, which falsely suggests that it might be meaningful to discuss whether, say, cognitive control and affect interact (Hommel, 2019a). However, the existence of unique labels for phenomena does not speak to the question whether the mechanisms underlying them are different or separable, and recent considerations indeed suggest that key functions of the human mind and brain make use of numerous unrelated, and not particularly dedicated characteristics that the human mind/brain has happened to generate through evolution: phylogenetic development has rendered the human brain increasingly selective, which means that the selectivity of information processing is a built-in feature of the modern brain rather than an achievement of a dedicated “attentional” mechanism (Hommel et al., 2019), and the concept of memory may collapse various unrelated and not particularly specialized characteristics of the human brain (Buckner & Schacter, 2004). Hence, the mere fact that we have invented a particular word to refer to one particular aspect of other people’s behavior does not guarantee that there is one dedicated system or module in the other people’s head that is responsible for producing exactly that behavior.

How can we avoid falling into the same trap when talking about goals? According to Braitenberg (1984), this might be achieved by replacing the more common analytical approach (from concept to subcomponent to system) by what he calls a synthetic approach. He argues that the top-down, analytical approach tends to generate too much theoretical overhead. As an example, he takes the study of Heider and Simmel (1944), in which naïve participants are presented with a movie showing geometric shapes moving across a surface. When the participants were asked to describe what they see, they substantially “enriched” the content of the movie by taking the shapes to represent people or objects and by inventing a story that provides the motivation for the observed movements. In other words, participants invented goals and intentions that helped them to organize and structure the complex movement patterns and attributed these goals and intentions to the shapes. Braitenberg (1984) argues that the theories that psychologists and cognitive scientists develop may often show the same tendency to over-interpret the observed behavior and, thus, the tendency to generate way too much theoretical overhead. As a cure, he suggests turning the research logic upside down and beginning with simple, well-understood mechanisms and trying to reconstruct the phenomenon under investigation.

Elsewhere, colleagues and I have argued that this may indeed be a useful strategy to simplify and unify theorizing in psychology and the cognitive sciences (Hommel & Colzato, 2015, 2017a), and I shall, therefore, follow the same strategy in this article. Hence, I shall suggest a parsimonious theory of how goal-directed behavior (i.e., behavior that both scientists and laypeople consider to be driven by what they call a goal) can be mechanistically explained. This theory will try to be as little original and rely on known assumptions about cognitive mechanisms as much as possible, with full credit to Occam’s razor. That is, the novelty of this contribution does not lie in the presentation of new assumptions about goals and their functionality but, rather, in the attempt to turn the available, mainly *descriptive* assumptions about goals (and *what* they do) into concrete, reasonably well-understood cognitive *mechanisms* (explain *how* they do that). Following the lead of Braitenberg (1984), my theoretical ambition will not consist in necessarily addressing all possible implications that the wide semantic field of the goal concept has to offer but, to the contrary, investigate how far I can get with as few new assumptions as possible.

As a platform, I shall use the Theory of Event Coding (TEC) that colleagues and I have suggested as a generic account of human perception and action planning (Hommel et al., 2001; Hommel, 2009, 2019b), which provides me with the basic representations and processes that goals need to operate on. While my present aim does not encompass the actual implementation of the suggested mechanisms, the fact that most of them can be implemented in working computational cognitive architectures that account for various phenomena and experimental effects has been demonstrated already (Haazebroek et al., 2017; Kachergis et al., 2014). Hence, GOALIATH is arguably less descriptive and circular, more transparent, easier to implement, and closer to meet Feynman’s challenge (“What I cannot create, I do not understand”) than other available cognitive, motivational, or social-psychological approaches to human goals. A first, preliminary sketch of the general idea I shall develop has been presented in Hommel and Wiers (2017) and Hommel (2019c).

## What is a goal?

In their comprehensive overview of the various ways, the goal concept is used in psychology and the cognitive sciences, Austin and Vancouver (1996, p. 338) “define goals as internal representations of desired states, where states are broadly construed as outcomes, events, or processes”. Very similarly, Heyes and Dickinson (1990) propose that a behavior can be considered a goal-directed action if it meets two criteria: the *belief criterion*, which is fulfilled if the agent shows evidence of knowledge about the relationship between behavior and eventual goal or outcome, and the *desire criterion*, which is fulfilled if the agent shows evidence of some sort of wanting the intended outcome. While both definitions seem rather basic, they can still be taken to reflect some unnecessary theoretical overhead. To explain in which sense this is the case, it is useful to follow Austin and Vancouver (1996, p. 339) in distinguishing between three analytical perspectives that goal theories reflect: the *latent perspective*, in which “goals define the pursuits of individuals, regardless of awareness or volition”, the *phenomenological perspective*, which focuses on how goal striving is perceived by the agent, and the *external-observer perspective*, which is interested in how external observers interpret the agent’s behavior in terms of meaningful goals.

In the following, I shall with very few exceptions ignore the phenomenological perspective. This may sound odd, as the phenomenal experience of goals, intentions, and desires has often served as the point of departure for theorizing about human action—especially in motivational and social psychology. Indeed, many influential theories segregate the process of engaging in goal-directed behavior according to criteria that directly reflect conceptual distinctions that are grounded in phenomenal experience. For instance, Heckhausen and Gollwitzer (1987) have suggested to distinguish four phases of action control: the *predecisional phase*, where potential goals are deliberated; the *postdecisional (preactional) phase*, where the agent implements the chosen goal; the *actional phase* that includes the actual action; and the *postactional phase* that serves for evaluating the achieved outcome. While the resulting distinctions may well relate to separable mechanisms or processes, there is surprisingly little evidence in support of the intuition that it is the phenomenal experience that is actually generating or triggering the underlying processes. Only very few, and rather minor aspects of action control have so far been related to conscious experience (for an overview, see e.g., Kunde et al., 2012) and even regarding these few aspects unequivocal evidence for a relevant causal role of phenomenal experience is still lacking (Hommel, 2007, 2013). These and other considerations have led Wegner (2002) to claim that conscious experience of intentional behavior may be *informed by* the actual causes of that behavior, but this information is always post hoc and presumably subserving more communicative purposes (Baumeister & Masicampo, 2010; Hommel, 2013).

More relevant for a mechanistic approach seems to be the latent perspective, which does not rely on conscious experience. However, when Austin and Vancouver (1996), and the authors they have reviewed, refer to goals as defining the pursuits of individuals, they take the existence of something in the head of the acting agent for granted. Note that this follows the typical research strategy of first identifying an interesting behavioral phenomenon (behavior that an observer can make sense of if considering it as reflecting a goal) and then concluding that there must be something specific in the behaving person that has no other purpose than generating the phenomenon (the attributed goal)—a kind of circular reification, especially if the attributed goal is taken to “explain” the observed behavior. But, just like in the case of attention, what we call goal-directed behavior may emerge from multiple constraints provided by different, perhaps even unrelated mechanisms that do not necessarily need to be dedicated to action control only (Schurger & Uithol, 2015). In other words, goal-directed behavior may be an emerging property of a human (or primate) brain, rather than the consequence of activating a particular mental representation or neural structure.

If so, the seemingly obvious plausibility of the latent perspective may actually derive from Austin and Vancouver’s third, external-observer perspective. Consider why even scientific researchers take the existence of internal goals for granted. Not unlike the participants in Heider and Simmel’s (1944) study, who spontaneously used high-level intentional concepts to describe translations of simple geometric shapes in a movie, we both as laypeople and as scientists find it useful to capture complex behaviors of others as expressions of internal goals. This allows us to make the description of the kinematic patterns of a hand movement steering towards a cup much more efficient by describing it as the expression of the goal to grasp the cup, and the description of the locomotion of a rat in a labyrinth much more efficient by describing it as an attempt to reach a particular goal location. However, we easily forget (as laypeople and as scientists) that the representation that our concept of a goal refers to is mainly in *our* (i.e., the observer’s) head, and whether it actually corresponds to something in the head of the observed agent is very much an empirical question—not a self-evident theoretical given.

These considerations raise the possibility that all behavior that looks to external observers like driven by the internal goals of the agent is actually a mere reflection of the agent’s cognitive infrastructure. Braitenberg (1984) gives a number of examples that render this possibility less far-fetched than it may seem. In his thought experiments, he constructs very simple vehicles made of just a few pieces of hardware that are connected in such a way that some input they are exposed to, such as a light source, makes them move in ways that Heider and Simmel’s participants would be likely to perceive as the acting out of particular action goals, emotions, and preferences. The purpose of these thought experiments is to demonstrate, as a proof of principle, that what observers would call goal-directed actions may very well be produced without anything that can reasonably be considered a goal, prediction, anticipation, desire, or intention. In principle, the same may apply to humans, which would reinforce Uithol et al. (2014) skeptical prediction that we may not find intentions in the human brain. And yet, three sets of empirical findings suggest that at least something we should be able to find.

The first set was obtained in the context of testing ideomotor theories of action control. Ideomotor theories assume that people continuously pick up all the sensory information about the effects their movements generate and associate this information with the motor pattern generating them. According to ideomotor theory, this amounts to the learning of possible action goals (Verschoor et al., 2010): once a particular motor pattern becomes associated with the codes of its sensory consequences, the agent can make use of these codes to reactivate the motor pattern intentionally, that is, to realize the now intended action consequences (i.e., the action goal) by endogenously reactivating the codes that spread activation to the corresponding motor pattern. Many studies testing ideomotor predictions were interested to see whether the execution of supposedly goal-directed actions is actually preceded by some sort of activation of codes that reflect the expected consequences of the action. Various observations suggest that this is indeed the case (for an overview, see Shin et al., 2010). For instance, lateralized stimuli were found to speed up the execution of actions with spatially compatible consequences (Hommel, 1993) and actions were initiated faster if their to-be-expected consequences were spatially or semantically compatible with them (Kunde, 2001). Brain-imaging studies provided converging evidence by showing that participants activate brain areas devoted to the processing of facial features or of non-facial body features before carrying out facial or hand movements, respectively (Kühn et al., 2011). Likewise, performing movements that generate the presentation of pictures of faces or houses is preceded by the activation of brain areas coding for faces or houses, respectively (Kühn et al., 2010). On the one hand, these observations do not yet demonstrate that the observed representations and activated brain areas play a causal role in the generation of the corresponding movements, as they may also represent causally irrelevant byproducts. On the other hand, however, they do show that agents code the expected outcomes of their actions and that the representations of these outcomes can affect and interact with action control. This would not be necessary if actions would merely be an emerging property of the agent’s cognitive system and is consistent with the assumption of observers that the behavior of agents is driven by some internal representation that in some way anticipates the action’s outcome.

The second set of empirical findings is related to cybernetic action-control models in the tradition of Miller et al., (1960; e.g., Blakemore et al., 2002, Wolpert & Flanagan, 2001). These models assume that agents create representations of the wanted or expected sensory feedback of actions so to evaluate whether an action was performed as intended or, if not, to assess the degree of discrepancy between expected and actual feedback. Studies motivated by these models have shown that the post-actional behavior of agents is systematically affected by the match or mismatch between the hypothetical expectations of the agent and the actual outcome of the performed action. For instance, if participants commit an action error, such as pressing the wrong key, they often spontaneously “repair” by pressing the right key right away (Rabbitt, 1966). Even if they do not, their performance is slowed down in the following trial, suggesting that they engage in some sort of error processing (Laming, 1968). Neuroimaging studies have again provided converging evidence, showing that post-performance brain activities differ systematically between correct and error trials (Bernstein et al., 1995). These and other observations would be difficult to understand if agents would not create some form of representation of what the action should look like, if they would not compare the expected and the actual outcome, and trigger internal processes that reduce errors in the following.

The findings obtained in the context of investigating ideomotor action control and post-error processing strongly suggest that agents form internal representations of the outcomes, events, or processes that their actions are likely to produce, which fits with Austin and Vancouver’s (1996, p. 338) definition of “goals as internal representations of desired states, where states are broadly construed as outcomes, events, or processes”. With one exception: the role of desire, which has also been highlighted by Heyes and Dickinson (1990), does not seem to be covered by the findings I have considered so far. The likely reason why authors feel the necessity to consider a concept like desire is the relative independence of human actions from the current stimulus conditions. At least the simpler ones of Braitenberg’s vehicles should react to the same stimulus conditions in exactly the same way, which certainly is not the case for humans—the third set of findings I have in mind. Numerous demonstrations that human behavior cannot be fully predicted by the present environmental state of affairs have led to the postulation of various kinds of concepts that are thought to account for this empirical variability, such as drives (Hull, 1943), needs (McClelland, 1988; Murray, 1938), motives (Schultheiss & Brunstein, 2010), motivations (Deci, 1975), desires (Reiss, 2000), or current concerns (Klinger & Cox, 2011). All these and other related concepts carry different kinds and amounts of historical and theoretical baggage, but they can all be understood as constraining the selection of actions in ways that can be considered to reflect a particular goal or a set of goals (Lewin, 1936). Most of these concepts comprise of one component that is relatively stable for a given agent, in the sense that some drives, needs, etc. are stronger or more expressed in some people than in others, and another component that varies in strength over time, such as hunger or thirst—to account for inter-individual and intra-individual variability, respectively.

To summarize, observers, including researchers of human cognition, have a long tradition of attributing the behavior they observe in acting agents to internal states in the heads of these agents that they assume are generating the behavior. While some of these attributions might be unnecessarily complex and producing too much theoretical overhead (Braitenberg, 1984), there are three empirical reasons to try saving aspects of the original goal concept: the observations that agents somehow represent the expected outcome of their actions, that they process and react to matches and mismatches between expected and actual outcomes differently, and that they can behave differently in the same situational context.^1^ How can we account for these three kinds of observations with minimal theoretical effort, so to avoid theoretical overhead, while at the same time providing concrete mechanisms that relate processes to the codes on which these processes operate?

## How is a goal represented?

A truly mechanistic theory needs to consist of assumptions related to processes, assumptions related to the codes or structures on which these processes operate, and assumptions connecting these two aspects (Hommel, 2020). In the following, I shall assume that it is this interplay between codes and processes that does whatever we mean when we assume that an agent “has a goal”. Hence, it is this interplay that I take to represent a goal. It is important to emphasize that this usage of the concept of goal representation does not assume any overhead, as common in many philosophically colored approaches, where representing a goal might imply having some understanding of the goal or some conscious awareness of it. I shall also avoid speaking of “mental [goal] representations” (as common in about 50% of the goal literature; e.g., see the contributions in Moskowitz & Grant, 2009), as the addition of “mental” must be considered either meaningless or pointing to a problematic dualist framework contrasting “mental” with “physical”. Hence, my usage of the term goal representation does not carry any theoretical ambition beyond the trivial expectation that the goal-directed character of a behavior must correlate with a particular interplay between internal codes and processes.

With respect to the codes and processes that represent a goal, our three sets of empirical observations provide some guidance where to look. The first set was obtained in studies investigating ideomotor models of action control. In essence, ideomotor models since Harless (1861) and James (1890) are based on the idea that the motor component of an action (e.g., action-specific neurons and neural patterns in planning-related compartments of the motor cortex) becomes associated with representations of the sensory outcomes of this action (e.g., neurons and neural patterns coding the action’s re-afferent feedback). Once created, this association can be used in the reverse direction: reactivating the representations of the sensory outcomes spreads activation to the motor component, which as a result leads to the now intentional performance of the corresponding action. Studies have suggested that the hypothetical bindings of motor patterns and outcome representations can become further enriched by representations of the particular situation, information about the accompanying affect, the task in the context of which the action is carried out, and other codes related to the entire action event (e.g., Dignath et al., 2019; Eder & Hommel, 2013; Spapé & Hommel, 2010; Waszak et al., 2003).

In the Theory of Event Coding (TEC: Hommel et al., 2001; Hommel, 2009, 2019c), bindings of this sort are called “event files” (Hommel, 2004), which represent the basic ingredient of TECs representational space. Hence, the theory denies any need to distinguish between stimulus and response representations, and considers the distinction between perception and action purely semantic in nature: Every event a perceiver/actor is exposed to is actively generated by the person herself, by moving her body and the respective receptors relative to her environment in a particular way, so that every perception is more or less actively produced and every action generates perceptual experience (Hommel, 2016). Calling this event a perception merely emphasizes the input processing activities of the perceiver/actor while calling it an action emphasizes the output production; and yet, these are just two, equally valid perspectives on the same event.^2^ For these reasons, TEC has no different compartments for perceptual experience and available action plans, but rather assumes that all cognitive activities operate on intermodal event files that subserve both perceptual and action-planning purposes, among other things.

**Fig. 1:**
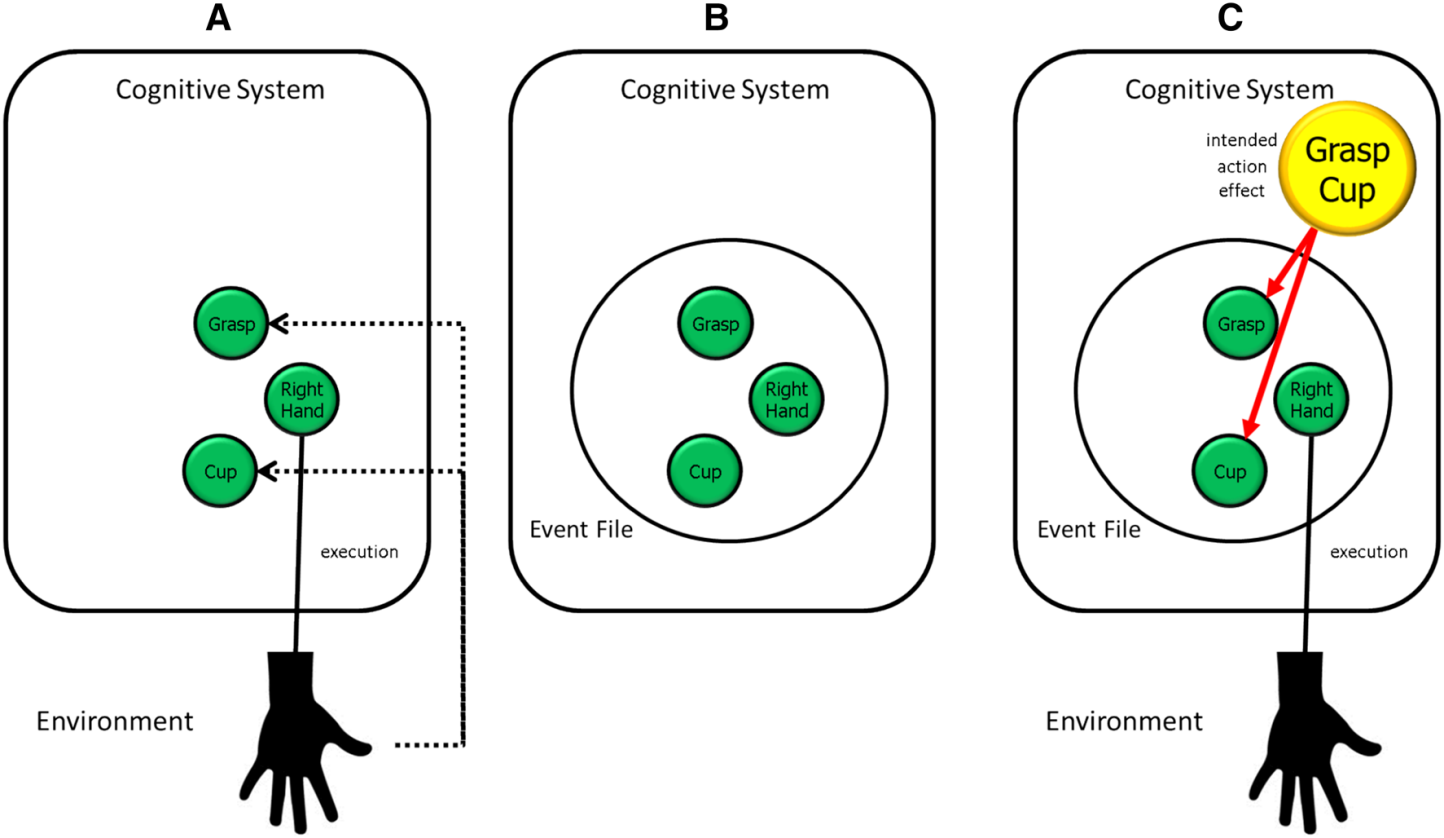
The basic idea underlying ideomotor action control. **A** captures the situation of the learner who carries out a cup-grasping movement for the first time, which according to ideomotor theorizing must have been non-intentional (and, thus, count as movement but not as action). The motor pattern controlling the movement of the right hand (Right Hand) is executed and still activated when the system receives re-afferent feedback about the movement and its consequences. This activates the codes corresponding to the respective features (Grasp, Cup in this example). Overlap in activation between motor pattern and feature codes leads to the formation of an event file (**B**), which now integrates the corresponding codes and tends to reactivate the entire event file upon activation of one or more of its components. This mechanism can then be used to carry out the action in an intentional way (see **C**): the agent imagines the intended action effect (Grasp Cup in the example), which in turn activates the corresponding feature codes in all available, or at least all contextually primed event files (only one file shown here). This leads to the eventual selection of one event file, which then controls the execution of the motor pattern

According to TEC, ideomotor learning creates event files that integrate the motor patterns underlying an action with the codes of this action’s sensory consequences.^3^ The perceiver/actor thereby acquires a representation that enables her to carry out the respective action in a voluntary fashion: she merely needs to “think of” a wanted action effect, which would activate all event files that contain codes of this effect, and which, thus, could be used to plan and carry out the associated action (see Fig. 1). Note that this scenario is sufficient to account for the observation that agents activate representations of expected action effects before performing the action. According to original ideomotor theory, the causally relevant process would be the “thinking of” activity that the agent engages in. However, not only would this line of thinking introduce a Rylean category confusion (as it would involve mixing personal and the systems levels), but it would also contrast with the already mentioned lack of evidence that conscious experience plays an important role in action control. It is, thus, more plausible to assume that the causally relevant process consists in the activation of the action-effect code, i.e., that part of the event file that represents the previously experienced sensory consequences of the action. This activation may or may not drive conscious experience as described by Wegner (2002), thus rendering conscious experience the consequence (Wegner’s *apparent cause*) rather than the cause of the activation (Wegner’s *actual cause*), but whether it does or not has no relevance for what follows.

If active anticipation is taken to indicate goal-directed behavior, and if the activation of action-effect components of event files can be considered to represent some kind of active anticipation, one can ask whether action-effect codes can be considered goals or goal representations. On the one hand, this might be an obvious choice and indeed a strong implication of ideomotor theorizing. This becomes obvious if one considers early development. The traditional view is that infants are born with particular goals but are in the beginning not yet able to sufficiently control their body and their effectors to reach them (e.g., Rochat, 2001). In contrast, ideomotor theory suggests that developing agents are acquiring possible future goals on the fly by interacting with their environment, which allows them picking up various action-effect contingencies (Verschoor et al., 2010). From this perspective, action-effect codes are indeed representations of possible and, if actively used, of actual goals. On the other hand, however, motivational scholars have argued that goals are necessarily general and insufficient to organize concrete actions, which presupposes the implementation of concrete action intentions (Heckhausen & Gollwitzer, 1987). From this perspective, goals would not be part of a structure that allows executing an action, as it is the case for event files. Action-effect codes would rather represent “intendable” effects, whereas goals would serve to select event files. To anticipate, I shall argue below that both views can be reconciled. In any case, we can conclude that even a generic framework like TEC provides sufficient cognitive infrastructure to account for the observation that agents anticipate the outcomes of their actions—without any additional theoretical assumption.

**Fig. 2:**
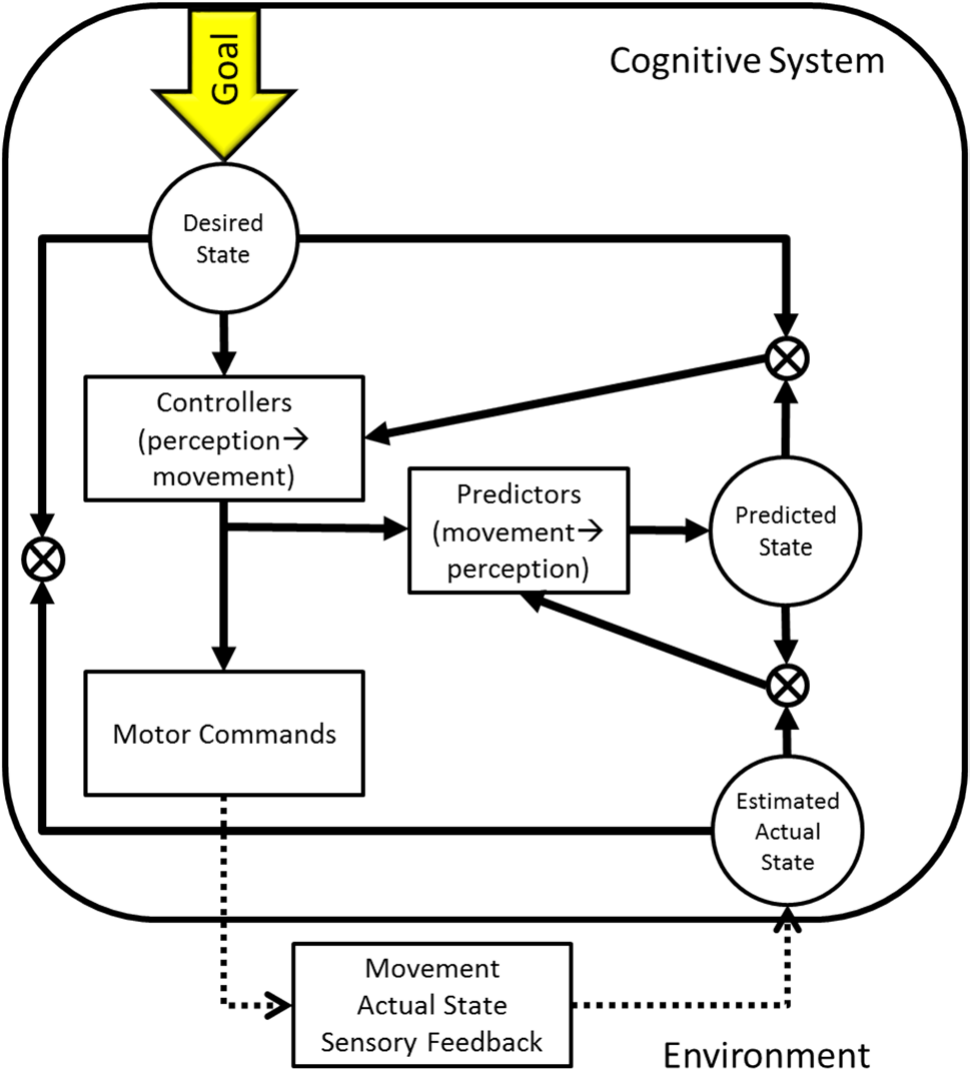
The basic idea underlying comparator models of action control, simplified after Frith et al. (2000), taken from Hommel (2017). The goal is defining the desired state, which controllers translate into motor commands that are eventually executed and predictions of expected outcomes. The re-afferent information is compared with the predictions, which results in an estimate of the discrepancy (the error, ranging from 0 in the case of perfect performance to higher values, depending on the degree of the failure)

The second set of observations suggesting a role of internal states in guiding what looks like goal-directed behavior has been obtained in the context of testing so-called comparator models and other cybernetic models in the tradition of Miller et al. (1960) and Powers (1973). A common assumption of these models is that goals activate both motor commands and representations of expected action outcomes. Once the motor commands are executed, the resulting re-afferent information is matched against the expected outcomes, which allows the agent to determine whether the intended goal was actually achieved. Ideomotor and comparator models differ in aims and emphasis, and they have different strengths and weaknesses (Hommel, 2015a; Verschoor & Hommel, 2017). Ideomotor theory strongly focuses on action selection and planning, as it tries to explain how the intention to realize a particular effect translates into activation and execution of motor patterns that eventually generate this effect. However, the theory does not have much to say about how the agent knows that the action was successful and performed as planned. In contrast, comparator models are very articulated regarding this action-evaluation part, which for instance has been suggested to play an important role in the experience of human agency (Blakemore et al., 2002; Frith et al., 2000). They are very silent, however, with respect to the question of how a given goal translates into a particular action and the movements needed to carry it out. Ideomotor and comparator models also differ in their emphasis on representations, which the learning-based ideo-motor models focus on, and processes, where the focus of comparator models lies. However, exactly because of these differences in perspective and emphasis, ideomotor and comparator models can be seen as complementary in terms of explanatory ambitions and suggested mechanisms (Hommel, 2015a; Verschoor & Hommel, 2017). Accordingly, it is tempting to assume that action-effect codes contained in the event files do not only serve the purpose of activating the event file that fits the currently intended action effect but also serve to create a reference against which the re-afferent information generated by executing the event file can be matched—just as comparator models suggest (see Fig. 2). Hence, the action-effect codes contained in event files are involved in two processes: action selection (emphasized by ideomotor theory) and action evaluation (emphasized by comparator models).

**Fig. 3:**
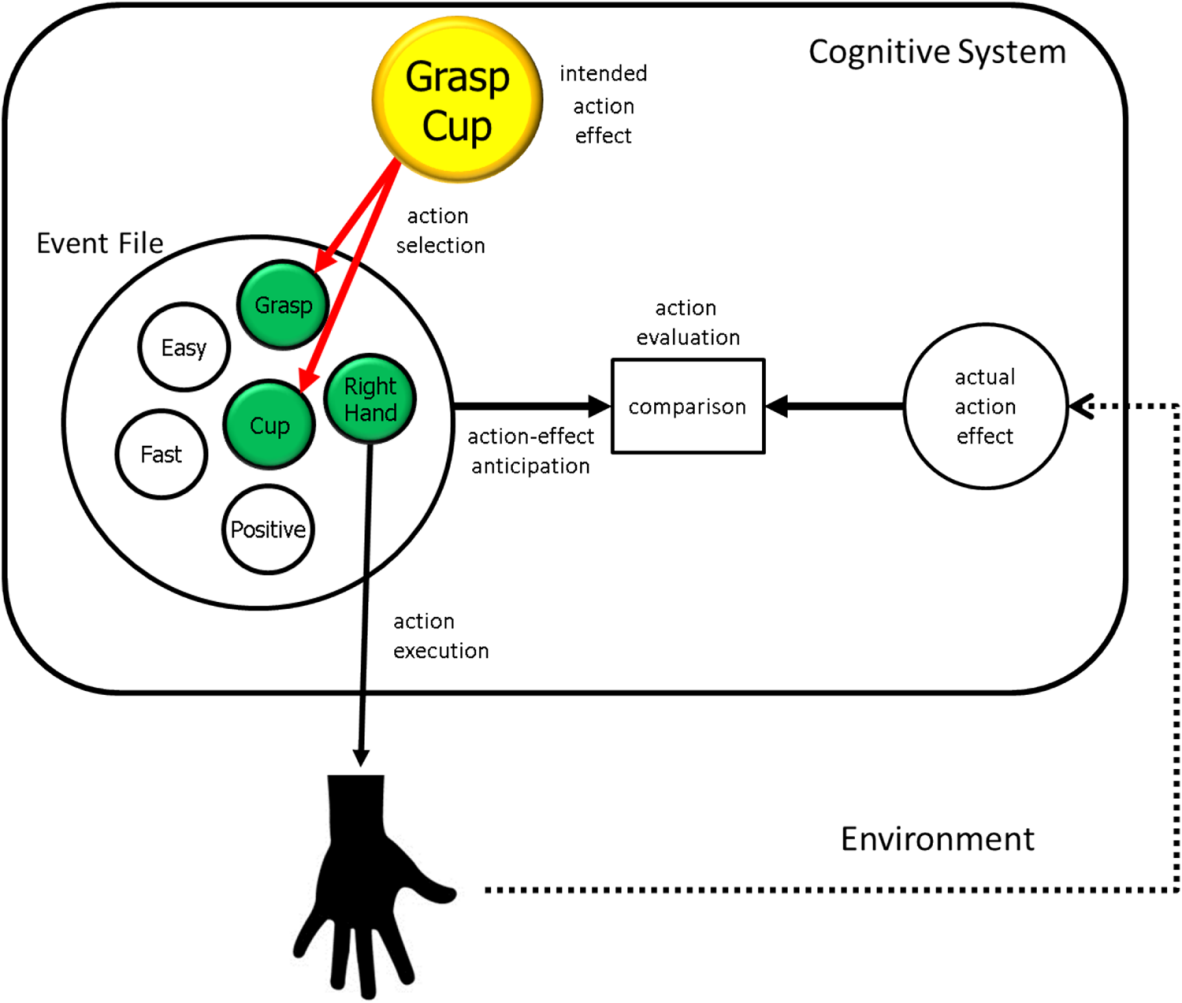
The basic architecture of an action-control model that integrates ideomotor theory and comparator models. Representing an intended action effect activates the matching feature codes in the available event files (only one shown here). Event files might contain more features, here representing the previous experience that the represented event was easy to perform, was fast, and led to a positive outcome, but in this example only two (Grasp, Cup) contribute to the selection of the event file. The match between intended action effect and corresponding feature codes in the event file is responsible for action selection. Activating the feature codes activates the entire event file, including the motor codes controlling the grasping movement of right hand, which leads to action execution. The re-afferent information from action performance is fed back into the system and compared with anticipated action effects (action evaluation). The outcome of this comparison is an error term that quantifies the degree of match/mismatch between anticipated and actual action effects

We can conclude that accounting for the first two sets of empirical observations with regard to what can be considered goal-directed behavior does not require any particular theoretical measures or any goal-specific extensions of existing theory. The observation of active predictions of the expected outcome in agents is fully captured by the basic infrastructure of ideomotor theory as incorporated in TEC. In particular, the inclusion of action-effect codes in event files that are assumed to underlie the generation of intentional action fully accounts for the finding that agents activate representations of expected action effects before the action producing these effects is carried out. The empirical evidence suggesting that agents compare expected and actual action effects is also captured by existing theory, as this comparison lies at the core of comparator models in the tradition of Miller et al. (1960) and Powers (1973), such as the more recent models of Blakemore et al. (2002). It is true that these and other insights suggest that ideomotor theory, with its emphasis on action selection, and comparator theory, with its emphasis on action evaluation, are complementary to a degree that calls for their integration into a comprehensive action-control theory, as suggested by Hommel (2015a). Figure 3 sketches the basic logic of how such an integration could look like. Note that the integration of ideomotor and comparator approaches successfully compensates for the weaknesses that these approaches exhibit in separation, namely, the lack of process assumptions in the former and the lack of representational assumptions in the latter.

While the first two sets of empirical observations are likely to tempt observers to conclude that the observed behavior is driven by some sort of internal state, they actually do not require the assumption that this internal state is well-characterized by calling it a goal. Even if we assume that the observed actions are initiated by activating an action-effect code that then primes the event file it is a part of, and even if this activation provides the basis for forming an expectation that is used to evaluate the outcome of the action, the initial activation may still proceed in a strictly stimulus-driven fashion. Even a strictly stimulus-driven action could be accompanied by an anticipation of its outcome and a test of this anticipation against the actual outcome—a test that predictive-coding approaches would consider necessary in order to avoid future surprises (Friston, 2012). One might save the goal idea by assuming that stimuli are not directly activating responses but goals that in turn organize appropriate actions (Watson et al., 2018), but these considerations would still not fully exhaust the semantic implications of the goal concept. However, extreme stimulus-centered views are inconsistent with the third set of observations, which suggests substantial inter- and intra-individual variability even under identical stimulus conditions. This is particularly obvious for basic biological needs: people eat more likely, and more, if they are hungry and they are more likely to drink if they are thirsty. But it also holds for acquired needs, as even the most affiliation-seeking individual needs periods of solitude and even the most performance-seeking person needs some rest.

Theoretically speaking, these kinds of variability are not sufficiently accounted for by a stimulus-driven approach, which raises the question why different event files can be activated under comparable environmental conditions. More specifically, we need to better understand how event files are activated in the first place. This is easy to explain in the context of typical experimental tasks, in which participants are instructed to carry out particular actions in response to particular stimuli. There is evidence that instructing participants to carry out action X in response to stimulus A and action Y in response to stimulus B is sufficient to create bindings between the representations of A and X and between the representations B and Y that are sufficient to automatically activate the responses by presenting the stimulus they are assigned to (Meiran et al., 2017). In other words, instructing people creates event files that link actions to stimulus conditions, so that these event files might be considered to represent the task goal. However, outside of the psychological laboratory, people commonly do not wait for particular stimuli to carry out instructed responses; the goal concept rather suggests that they choose actions to realize their internal goals.

From an ideomotor perspective, formulating an internal goal should consist in activating a representation of an intended effect. However, goals and effects can be described in various ways and at various levels (Vallacher & Wegner, 1987): an agent may refer to the very same action as quenching her thirst, having a soft drink, reaching for a particular glass in front of her, moving it to her lips and drinking from it, etc. Interestingly, there is evidence that these are not just semantic choices in communication but apparently reflections of different ways to control the action. Vallacher and Wegner (1987) provide a comprehensive review suggesting that agents keep monitoring their action at a particular level of description, systematically gravitate towards the more molar description level if different levels of description are available, and move to the lower levels if something goes wrong. Converging evidence was reported from studies of typewriting (Crump & Logan, 2010) and of handwriting, where the level of control systematically varies with practice, moving from the control of individual strokes to the production of letters and, later, words (Hulstijn & van Galen, 1988). From an ideomotor point of view, this means that intended action effects can be specified at various levels that differ in detail. The less specific an intended action effect is represented, the less likely is there just one event file that contains the code of this effect. Rather, increasing the general specifications should activate increasing numbers of matching event files that compete for selection.

**Fig. 4:**
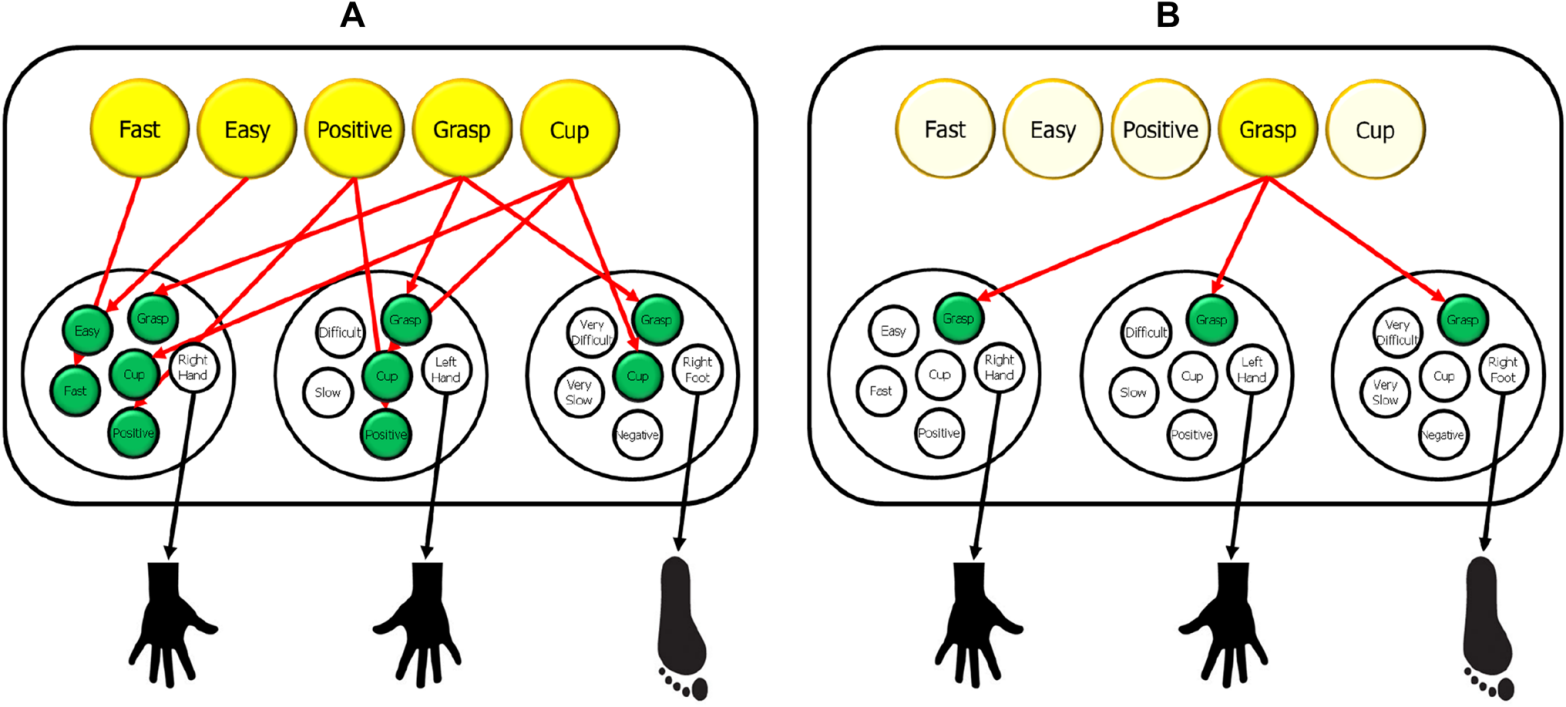
The implications of concrete and abstract goal definitions. **A** Shows a situation in which the goal criteria specify the sought-for action as one that should result in grasping a cup (in a particular location, not shown) as fast as possible, with only little effort, and in a way that guarantees a positive outcome (an example taken from Hommel & Wiers, 2017). In this example, these criteria activate event files related to actions with the right hand, the left hand, and the right foot. The right-handed agent is assumed to have experienced that all three actions can be used to grasp a cup, but the right-hand action clearly matches more goal criteria than its competitors. **B** Shows a situation in which the goal is much more abstract, namely, to grasp something. In this case, all three event files show a match but without any clear winner. The eventual selection will thus be a random choice between the three candidates, which implies much more variability in choice outcomes over time

In the example shown in Fig. 4 (following the logic of Hommel & Wiers, 2017), an agent is planning to either grasp a cup as fast and effortless as possible and with a high degree of accuracy (i.e., a high probability of a positive outcome) (Panel A) or to just grasp something (Panel B). It is obvious that the more abstract definition of the intended action effect creates stronger competition by activating more equally active event files. The more competition exists, the less likely will repeated selection of one suitable event file favor the same file, which implies that more abstract definitions of intended action effects create more space for inter-individual and intra-individual variability. Note that this is a strongly simplified scenario, because other factors are also likely to affect event-file selection. For instance, repeating one action in the same context is likely to render the representation of this action more context specific, which in turn will increase the probability to select this event file in the corresponding context. Another example is practice, as increasing practice of one particular action will facilitate the performance of this action. The greater ease of this action will be coded in the event file and favor selection of this file, depending on the emphasis on saving effort by the agent. Nevertheless, all other things equal, more abstract definitions of intended action effects will lead to greater variability. Moreover, as I shall discuss in the next section, there are reasons to believe that the kind of action-effect specifications can vary over time, which will also contribute to variability.

While the introduction of criteria for event-file selection is sufficient to account for the third set of empirical observations, it does represent an extension of the original TEC. As criticized by Cisek and Kalaska (2001) and discussed by Hommel (2019b), the original TEC was mainly interested in describing the representational infrastructure of the human cognitive system and the key processes that operate on the postulated representations; control processes that tailor this infrastructure to the current situation and task were widely ignored, however. This has been repaired in more recent contributions (Hommel & Wiers, 2017; Hommel, 2018, 2019b), which also addressed how event files become selected. For present purposes, it is important that the goal-related implications of the third set of findings can be fully covered by assuming that the criteria used to select event files are often sufficiently general to allow for competition between multiple event files, and that the kind of criteria being used is likely to vary over time (see below).

## How many goals do agents pursue?

Motivational theorists have established a long-standing tradition according to which the production of goal-directed behavior falls into two different phases: goal-setting, which represents the actual motivational aspect, and goal-striving, which represents the volitional aspect of action control (Gollwitzer, 1990). The process of goal-setting is commonly conceived as the battle of drives, needs, motives or whatever the theoretical terminology for the driving forces might be, that settles into a solution that leaves only one goal. What follows is the process of goal-striving, which is conceived of all the processes that are involved in translating the goal into actual behavior. The transition between these two phases has been compared with Cesar’s crossing of the Rubicon (Heckhausen & Gollwitzer, 1987) that turns mere wishing into actual intending, and the two phases have been shown to be associated with different mindsets (Gollwitzer, 1990) or modes of information processing.

On the one hand, these scenarios could be easily translated into the theoretical assumptions we have developed so far. Driving forces like hunger and social motivation may make the decider struggle between preparing a meal and joining her friends in going to the cinema, before she eventually invites her friends for dinner. This process may start with specifying criteria like “hunger-reducing” and “socially interactive”, which first activates all event files that satisfy at least one of these criteria and eventually results in the strongest activation of the event file that fits best with these criteria. The eventual selection of the most active event file would represent the crossing of the Rubicon and, thus, the transition from goal-setting to goal-striving. On the other hand, however, this example leaves open how we can define the actual goal: were there two goals (reducing the hunger and satisfying the need for affiliation) that continue to coexist or does the solution integrate two different goals into one? How do we define goals and how do we count them?

This may look like an academic question of little consequence, but it raises further questions regarding theories of action control and, in particular, goal-directed action selection. For one, while few cognitive theories address this question explicitly, many implicitly assume that only one goal can be active at one time. For instance, most theories of attentional selection consider the processing of unpredictable information that is unrelated to the instructed task a failure or breakdown of attentional control, such as when participants process the color word in a Stroop task (1935), the symbols flanking a target symbol (Eriksen & Eriksen, 1974), or a visual oddball (Theeuwes, 1992). At the same time, humans are suspected to be notoriously curious (Berlyne, 1960), consistently exploiting statistical regularities of their environment (Barlow, 2001), and driven to eliminate uncertainty (Parr & Friston, 2017)—which would render it odd if they would not spend at least some attention to stimulus events that may well be related to their task. Hence, the claim that the processing of nominally irrelevant information demonstrates a lack of attentional control directly implies, and in fact hinges on the implicit assumption that instructing participants of a Stroop, flanker, or visual-search task effectively suspends basic drives of higher evolutionary importance. That this implicit assumption is likely to be incorrect is suggested by recent findings showing that reducing the uncertainty regarding the nominally irrelevant information (i.e., reducing the degree to which this information satisfies the curiosity drive) leads to a drastic reduction of the impact that this information has on action control (Frings et al., 2019; Hommel et al., 2021).

Most theorizing about executive control is facing similar logical problems. In particular, some theories implicitly or explicitly assume that pursuing and maintaining a given action goal against all odds is an indication of well-functioning cognitive control. Accordingly, each indication of reduced maintenance and shielding is interpreted as a control failure (e.g., Botvinick et al., 2001; Kool et al., 2010; Miller & Cohen, 2001). Other theories, however, consider that optimal control always tries to find a balance between goal maintenance and openness to goal change (e.g., Cools & D’Esposito, 2010; Dreisbach & Goschke, 2004; Hommel, 2015b). For instance, studies of reward learning have used a multi-arm bandit design in which participants have the opportunity to select the bandit machine that they believe to generate the highest profit (Daw et al., 2006). Performing such a task requires repeated decision-making whether to stay at the same machine or to switch to a new one, which implies that participants need to constantly decide whether they should maintain their current goal or switch to another one (Cohen et al., 2007). One may try avoiding this logical inconsistency with the maintenance principle used in other accounts by considering outcome optimization the actual goal and maintenance or switch a subordinate decision under this goal. However, this theoretical move overlooks that participants of less complex experiments face the exact same problem: should they really spend more minutes on a boring, repetitive reaction time task for just a few credit points, or would they not be better off leaving and spending the rest of the day with more interesting activities? Hence, temporary reduction in the enthusiasm to carry out a demanding artificial task for little reward, as thought to be indicated by task-switching costs (e.g., De Jong, 2001) or compatibility-sequence effects (Stürmer et al., 2002), may not so much reflect a loss of control but, rather, a brief glimpse of rationality that takes more interesting and more rewarding goals into account. If so, instructing a participant to carry out an artificial task could not be assumed to switch off other goals and interests that she is likely to bring to the lab.

The emerging picture fits with the framework of Atkinson and Birch (1970), who suggested that agents are facing a continuous, dynamic struggle between alternative action tendencies (a scenario later also propagated by Kruglanski et al., 2002). Contrary to what the Rubicon metaphor implies, this struggle need not stop when decisions have been made and intentions have been implemented.^4^ For instance, even if participants receive a fully valid precue which action they are to carry out in response to the next stimulus, they perform faster if the location of the stimulus is compatible with the location of the response (Hommel, 1996). Given that stimulus–response compatibility is assumed to target response selection (Kornblum et al., 1990), this suggests that having made the decision to carry out a particular response does not shield it from information suggesting other responses. Likewise, moving one’s hand towards a particular goal location is still affected by the presence of other possible goal locations long after the movement has been initiated (Hommel et al., 2017). Observations from action-error studies provide further evidence for ongoing dynamics: as already mentioned, incorrect responses are often spontaneously corrected, suggesting that “deliberation” went on even after the incorrect response was selected. The concurrent activation of multiple goals is also obvious from congruency effects in task-switching studies, which indicate that responses in the current task are performed faster and more accurately if the corresponding response would also be correct in the alternative, currently irrelevant task (Meiran, 1996). Along the same lines, performance on the primary task in dual-task designs is better if the corresponding response is compatible with the response related to the following, secondary task (Hommel, 1998). Hence, the idea that all that matters for participants of laboratory tasks is the currently relevant task goal seems to be unrealistic.

Related unrealistic assumptions have been put forward by various models of, or with implications for, action control, by postulating that actions are selected to comply with single optimization principles. For instance, it has been suggested, and demonstrated in numerous studies, that action selection is optimized for picking the least mentally costly (Kool et al., 2010) or effortful (Rosenbaum et al., 1995) action, the action that provides the most information about one’s environment (Friston, 2012) or the most rational solution to a problem (Kahneman, 2011). As it is unlikely that all of these principles point to the same action, we need to conclude that either all but one of these suggestions are wrong or they all cover only part of the truth. If each suggestion points to a separate driving force, whether we call it a drive, need, or goal, and each of these forces contributes selection criteria to the action-selection process, we need to consider action selection a multiple-constraint-satisfaction process that tries to satisfy many, partly inconsistent goals at the same time.

Importantly, these considerations imply that goals do not represent coherent entities that can be located in a systematic goal hierarchy, as suggested by various approaches (e.g., Koechlin et al., 2003; Vallacher & Wegner, 1987). Goal hierarchies are considered hierarchies because they postulate a particular logical structure, in which components at lower levels have lower complexity and make up or embody components at higher levels, like going on vacation consists of the subgoals packing one’s bags, loading one’s car, navigating it towards the goal, etc., where packing one’s bags consists of further subcomponents that consist of further subcomponents, and so forth (Uithol et al., 2012). Our previous considerations suggest a much less structured, more eclectic combination of goal components. Rather than following the semantic or practical decomposition of activities, it seems to be possible that goals consist of unrelated criteria used to select event files. For instance, it seems to be possible to merge the goal of packing one’s bag, say, with the situational requirement to do that fast, to do it together with another family member, and to do that by optimizing spatial resources, even though each criterion may be driven by a different goal.

This idea of goals as referring to composites of (possibly) unrelated selection criteria is also suggested by the literature on process priming (Janiszewski & Wyer Jr., 2014). While the replicability of some findings in this area is under discussion (e.g., Doyen et al., 2012; Harris et al., 2013; but see Diksterhuis, 2014, and Stroebe & Strack, 2014), there is at least some evidence that people can be primed to carry out particular actions or actions in a particular way without telling them explicitly to do so. For instance, participants perform faster after having shadowed a speech that required them to talk rapidly (Shen et al., 2012) or after having been presented with names of fast-moving animals (Aarts & Dijksterhuis, 2000). Importantly, the tasks in which the faster performance was measured were different from and unrelated to the tasks that were inducing the priming. This suggests that it was unlikely to be integrated task goals that were primed but, rather, only specific task parameters or, as the theoretical scheme I am developing here would suggest, independent goal criteria that were considered in selecting the appropriate event files.

Taken altogether, it seems unrealistic to assume that agents entertain single, definable, and coherent goals, organized into recognizable hierarchies, that they translate into concrete action in an orderly sequence of phases. Rather, they seem to use composites of possibly unrelated and possibly contradictory selection criteria to favor the best-matching event file. Event-file selection does not seem to consist of a discrete act that stops the dynamic matching process, so that the preferred event file can change over time and impact overt behavior continuously.

## Selection criteria select event files

The Rubicon metaphor and its distinction between goal-setting and goal-striving seem misleading in implying some unidirectional, discrete act that converges onto one single action tendency subserving one single goal. One may object that the distinction between selection criteria on the one hand and a selected event file, and its further impact on action control, on the other seems rather similar to the distinction between goal-setting and goal-striving, respectively. However, going somewhat further into the details of the event-file selection process refutes this objection and reveals further misleading implications.

Let us begin with the question where the hypothetical criteria for selecting event files come from. The most obvious choice might seem the instruction given to the agent. Cognitive research draws upon the remarkable ability of human participants to do what they are told, that is, to reconfigure their cognitive system in such a way that they are able to carry out almost any arbitrary task. While it is still a mystery exactly how participants translate instructions into tailor-made task-sets (Brass & de Houwer, 2017), it is clear that they master this skill, possibly by creating ad hoc associations between representations of stimuli and responses before encountering the particular events (Meiran et al., 2017). This implies that stimuli can become associated with criteria that refer to a defining feature of the assigned response. For instance, an agent who is to respond to a green stimulus by pressing a left key (and to a red stimulus by pressing the right key, say) would create an ad hoc association between the code of the feature green (Green) and the code Left, which in turn would be used as a criterion to select the event file that is driving the correct response. Seeing something green would, thus, activate the criterion Left, which in turn would activate all event files that include the code Left. Given that adult agents are likely to have thousands of event files that include this code, selecting one event file would take much too long for experimental purposes. However, given that instructions commonly specify the response set, that is, the characteristics of permissible responses, participants are likely to have the left and right key press prepared—meaning that they have increased either the base level of activation for the two corresponding event files or the weighting of input to these files (Memelink & Hommel, 2013). Accordingly, only the match between these two files and the selection criteria would matter, so that activating the criterion Left activates only the event file driving the left keypress.

Another obvious source of selection criteria are basic biological drives like hunger or thirst. Agents commonly bring a history of experiences in which these drives were successfully reduced, and the ways they were reduced will share some similarities: reducing hunger will likely have to do with food, reducing thirst with particular kinds of liquids. This will have resulted in bindings between the sensory features of experiencing hunger (e.g., feeling a “hole” in one’s stomach), the features that refer to hunger-reducing characteristics of possible food (e.g., being edible, looking tasty), and the features of actions that drive approach and eating behavior (e.g., reducing the distance between agent and food, bringing the food object to one’s mouth). If so, perceiving that one is hungry would activate the criteria Edible, Tasty, Approach, Bring to mouth, among others, which would increase the probability of selecting event files that include these criteria, and eventually select the file that does so best.

This rationale is not restricted to the nature of the drive. While basic needs like hunger or thirst are part of our shared human hardware, other needs have been argued to be more strongly shaped by upbringing and personal experience. McClelland (1988) assumed that parents, peers, and other social context establish particular themes that play a major role in seeking particular kinds of reward for particular kinds of actions. The *need for achievement* is one, characterizing individuals who were raised with an emphasis on autonomy and high performance. Another is the *need for power* and the *need for affiliation*, characterizing individuals who learned to receive their reward from dominating or teaming up with others, respectively. Depending on one’s individual need profile, an agent is, thus, likely to prefer actions with the feature of showing her performance, increasing her power, and/or bringing her in closer contact with others, suggesting that one’s personal need profile is associated with the enduring activation of selection criteria that specify the respective features and, thus, favor event files with features that match. But even McClelland’s approach is not exhaustive, and other authors have suggested important roles of other needs, such as the *need for closure* (Kruglanski & Webster, 1996).^5^

Acquired needs may be somewhat less stable and dominant than basic, survival-relevant biological needs, but both kinds of needs can be assumed to impact action selection in ways that are not different from the function of short-term goals: the current strength of the drive or need determines the degree to which the associated selection criteria (favoring event files with action effects that satisfy the need (see McClelland, 1988), like showing one’s achievement or enhancing one’s power) impact event-file selection. Hence, the stronger the drive/need the more likely the agent prefers event files that are likely to generate action effects that reduce the drive/need (i.e., the more likely an event file is selected that contains a drive/need-reducing action-effect code). This scenario basically represents an application of Lewin’s (1936) concept of quasi-needs. In trying to understand how goals can drive intentional action, he claimed that simple action goals function exactly like drive- or need-instigated behavior. Setting a goal, Lewin reasons, creates a discrepancy between what the agent wants and what actually is the case, and the degree of this discrepancy determines the amount of motivation to reduce and eventually eliminate it. Hence, short-term goals lead to the establishment of selection criteria, just like drives or needs (i.e., more chronic goals) do, so to eventually select the event file that matches these criteria best, which in turn is likely to result in actions with effects that satisfy the goal. Hence, biological drives, acquired needs, and adopted goals may differ in origin, stability, and strength, but they are comparable in terms of mechanisms: they are associated with selection criteria that bias the action selection towards actions satisfying them.

To summarize, multiple sources can contribute various kinds of selection criteria that serve to favor event files with features that match these criteria, with the best-matching event file to be the most likely to be eventually selected. This means that short- and long-term goals are represented in terms of (sets of) selection criteria that functionally correspond to reference values in cybernetic theories like those of Miller et al. (1960) and Powers (1973) (Austin & Vancouver, 1996). Importantly, selection criteria are likely to be provided by various factors that differ in origin and purpose, and their relative contribution at any point in time will depend on their support and strength of activation but not on their relationship to other factors. This means that event-file selection does not consist in translating a single, coherent goal into a specific intention but rather emerges from the attempt to satisfy multiple, unrelated constraints and requirements. If goals consist of a patchwork of selection criteria, it makes little sense to speak of anything like “a” or “one” particular goal that is driving the behavior of an intentional agent.

## The format of selection criteria

So far, our discussion suggests that goal-directed behavior is driven by multiple selection criteria, which in turn consist of feature codes that match the action-effect codes contained in event files. These feature codes need to be active to some degree to impact the selection process, and we have seen that the causes of when and why they are active may differ in various ways. That people keep and store codes of event features is a key postulate of TEC and supported by many empirical observations. And that codes need to be activated to matter seems to be a reasonable assumption to make as well. But is that all it needs? Can any activated feature code serve as a criterion? Do feature codes have to be of a particular format to impact action selection?

While these issues still await any systematic theoretical and empirical treatment, some authors have suggested particular kinds of formats that effective feature codes should have. For instance, even though James (1890) was more interested in kinesthetic effects, the central role of ideomotor theorizing in explaining imitation (Prinz, 2005) might be taken to suggest that agents often use visual imagery in controlling their actions. More recent approaches have focused on the role of what is called “motor imagery”, which is assumed to play a key role in action planning (Decety, 1996). Unfortunately, the term is a misnomer because it does not refer to the modality of the information on which the imagery is based (as with “visual” or “auditory” imagery) but to the activity that is being imagined—thus begging the question in what format or modality the imagery process takes place (which may still be visual or auditory, or perhaps proprioceptive and kinesthetic, the two modalities closest to action). Other authors, like Luria (1961) and Vygotsky (1962) have considered verbal labels of actions and action effects as particularly effective, as early development shows some interesting parallels between increasing expertise in intentional action planning and systematic changes in accompanying verbal self-descriptions (see below).

The lack of systematic empirical research on this issue does not allow for strong conclusions, but it is fair to say that so far there is no evidence for any privileged impact of particular kinds of formats. For instance, studies on the acquisition of novel action effects have found effects with visual (Kühn et al., 2010), auditory (Elsner & Hommel, 2001), and tactile action effects (Wirth et al., 2016), and with both verbal and non-verbal effects (Koch & Kunde, 2002; Kunde, 2001), which does not suggest any privileged modality or format for action-effect coding. There is also evidence that the acquired effect codes do keep information about their specific sensory origin, as can be seen in effects of modality compatibility (e.g., Földes et al., 2017; Hommel & Müsseler, 2006). An unpublished study from our lab (Prokofieva, Schaefer & Hommel, unpublished) suggests that musicians playing different instruments prefer coding their musical actions in matching modalities (i.e., stronger visual and weaker kinesthetic preferences in piano players than in violin or woodwind players). Learning experience might, thus, induce particular preferences, at least in particular tasks, but does not seem to be a privileged modality or format for coding actions or selection criteria.

**Fig. 5:**
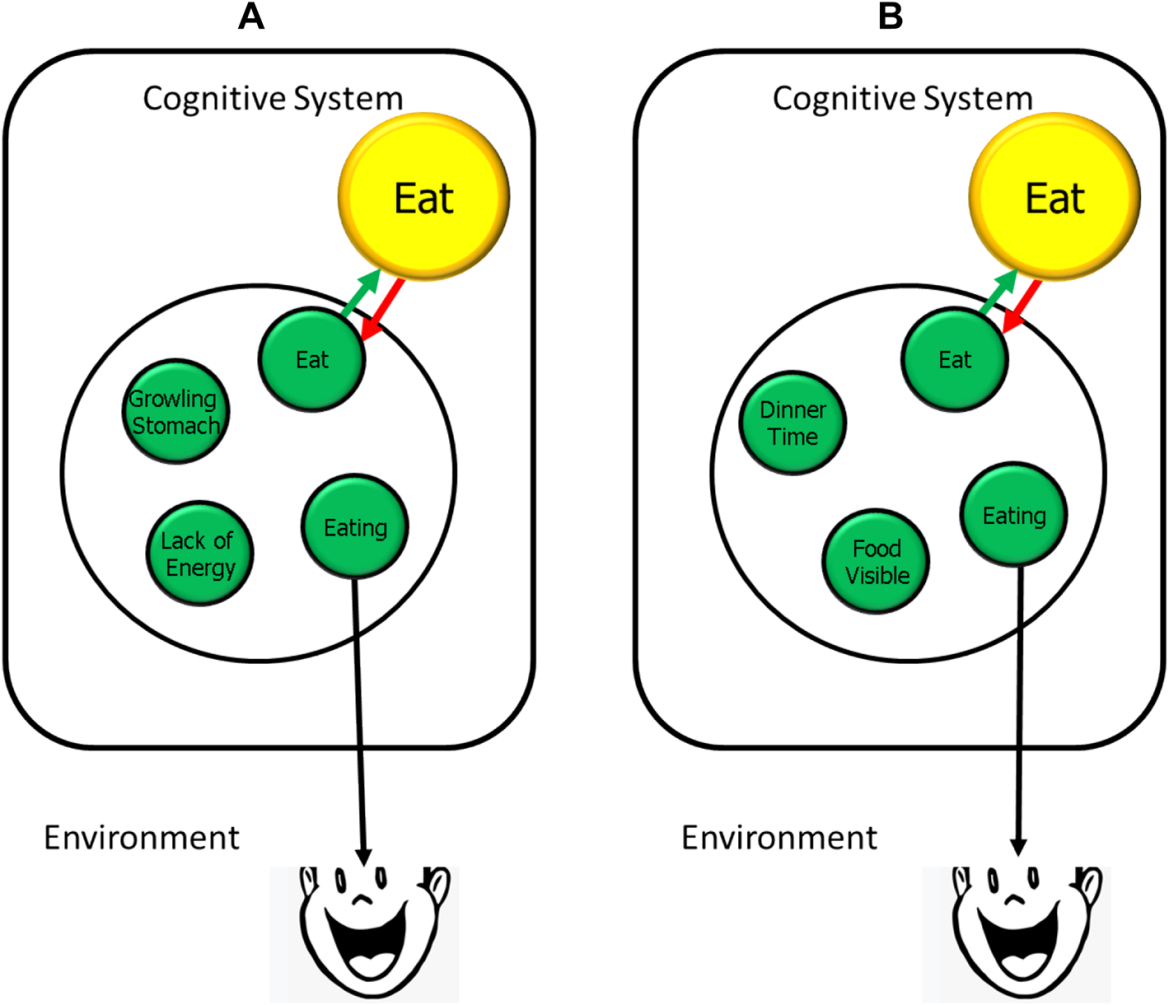
The impact of individual differences in interoceptive perception on eating (i.e., hunger-reducing) behavior. **A** Characterizes the representation of eating in an individual with good interoception. The motor act of eating (or of any hunger-reducing activity in general) is likely to co-occur with the person perceiving herself to eat, to have a growling stomach, and a lack of energy. Over time, the binding of the respective codes will become the default representation of eating. Accordingly, perceiving one’s stomach to growl and having a lack of energy will activate the Eating-file(s), so that the Eat feature will get activated. It, thus, can serve as a goal criterion to select eating-related event files. Note that the green and the yellow Eat code are actually the same, the color only indicates the particular function as member of an event file on the one hand and as highly active feature code that serves as a selection criterion on the other. The difference in color does, thus, not indicate any particular format or location of the respective feature. **B** Shows the representation of eating by a person with less optimal interoception. The act of eating and the self-perception thereof will be less likely to co-occur with internal activities and the signals they produce but, rather, with external events, like dinner time or visible food. It is, thus, these external events that are likely to trigger eating behavior

It may sound odd to assume, as I do, that any sufficiently activated feature code can contribute to action selection, so that the ingredients of event files can act as selection criteria only because of their degree of activation and, thus, bias the selection of other event files (see Fig. 5 for an example). While this would explain why and how effects of process priming (Janiszewski & Wyer Jr., 2014) occur, it may seem more obvious to reserve a particular action-planning system to holding codes that are used to control one’s intentional actions. For instance, Miller and Cohen (2001) have suggested that information that represents the action goal and that is used to provide top-down guidance for action selection—which is pretty much the function that I have ascribed to selection criteria—need to be loaded into a particular functional system housed in the prefrontal cortex. As the prefrontal cortex is unlikely to be the default location of feature codes based on sensory information (see Kühn et al., 2011), this suggests that, in order to be effective, feature codes would need to be copied or connected to a dedicated system that, according to Miller and Cohen, is strongly capacity limited. A similar proposal was made by authors working with ACT-R (e.g., Anderson & Lebiere, 1998), which holds that the current goal is held by a dedicated, capacity-limited system that is guiding lower-level cognitive processes. If human goals would really be limited by such capacity limitations, it would be difficult to see how multiple goals or goal criteria could be maintained.

The reason underlying the apparent discrepancy between the approaches of Miller and Cohen or Anderson on the one hand and my present suggestion on the other becomes clear if we consider Cowan’s (1995) integrated memory model. According to Cowan, working memory or short-term memory (two terms the author considers practically identical: Cowan, 2008) consists of all sufficiently (i.e., above-threshold) activated elements of long-term memory. The activation can come from various sources, including current stimuli, leftovers from previous cognitive activities, ongoing thoughts, etc. A not-further-described central executive adds (capacity-limited) activation to a few elements of particular current interest, which forms the “focus of attention”. Importantly, all activated elements are assumed to have an impact on action selection, irrespective of whether they are in or out of the current focus of attention. This fits with my suggestion that the degree of activation of feature codes is all that matters for having an impact on event-file selection.

However, Cowan draws a distinction between actions that are affected by activations that are supported by the central executive (“controlled actions) and actions that are affected by activations falling outside of the focus of attention (“automatic actions”). If we consider the fact that Miller and Cohen (2001) and Anderson and Lebiere (1998) are exclusively interested in the production of what Cowan calls “controlled actions”, it becomes clear that the goal-holding system that Miller and Cohen or Anderson envision does not exhaust working memory but restricts itself to Cowan’s “focus of attention”. On the one hand, such a distinction makes sense because arbitrary goal criteria, as instructed in typical laboratory tasks, do not enjoy the relatively stable support by biological drives, acquired needs, or other kinds of current concerns (Klinger & Cox, 2011) that more natural, personal goal criteria receive. It, thus, seems plausible that some extra cognitive work is required to make them reach equal status and provide them with the same or even stronger impact on action selection than their natural competitors. That this extra work is carried out by the same cortical system that is also responsible for other meaningless operations, like the maintenance of arbitrary number sequences (a typical working-memory task), makes a lot of sense. On the other hand, however, claiming that it is this extra cognitive work and this contribution from prefrontal systems that renders an action truly goal-directed, and to discount actions that are driven by the more natural interests of the agent as non-goal-directed and “automatic”, seems to overestimate the degree to which arbitrary translations of sets of meaningless stimuli into sets of meaningless keypresses represent human action control.

To summarize, there is no evidence that action-effect codes need to be of any particular kind or format to function as event-file selection criteria or that criteria need to be represented in particular functional or cortical systems dedicated to representing human goals. However, it is likely that criteria that are provided by, or associated with longer-term drives, needs, or other kinds of interests require less cognitive support to impact event-file selection than arbitrary ad hoc criteria without any connection to the agent’s real-life desires.

## Goal dynamics

If it is true that goal criteria that are in agreement with the longer-term interests of an agent require less cognitive support to make them competitive in the selection of event files, we need to ask how this can be the case. According to Atkinson and Birch (1970), action tendencies continuously change in their activation level, which implies that the selection criteria that I claim to modulate the activation level of these action tendencies should continuously change in activation as well. So, what is it that determines the activation level of action criteria, even in the absence of external instruction, and what makes the selection criteria that relate to our natural desires so particularly sticky?

This is easy to understand in the case of biological drives. The physiological signals of hunger, thirst, and other drives vary over time, depending on glucose levels in the blood, insulin and leptin levels, and more. If these signals, or their perceivable consequences like stomach growling or a lack of energy, become bound to feature codes that act as selection criteria for event-file selection through Hebbian learning, the degree to which the feature codes actively impact event-file selection systematically covaries with the level of the corresponding drive. That is, the hungrier I am, the more strongly activated will the associated selection criteria be, and the more this will bias my action selection towards event files with hunger reduction as one of their action effects.

However, the correlation between physiological signals and the activation of feature codes is likely to vary from individual to individual. Schachter (1971) suggested that individuals differ with respect to their sensitivity to internal signals, such as those related to physiological hunger, an idea that has also been entertained with respect to emotions by Laird (2007) and Bermond et al. (2010). The lower the sensitivity to internal signals is, the more the individual will rely on external signals, such as time of day or availability of triggering stimuli, like food. As a consequence, people may not necessarily eat when they are physiologically hungry but when it is dinner time, say, which among other things may lead to obesity (Schachter, 1971). For our present purposes, this implies that feature codes are not necessarily bound to the signal that makes most physiological sense but to the signal that is actually used to trigger a particular behavior, such as the time of day and the visibility of food in some individuals and being close to the fridge in others (see Fig. 5).

It would be tempting to consider acquired needs, like McClelland’s needs for achievement, power, and affiliation, to be responsible for driving their associated feature codes just like biological drives do. In the literature, acquired needs are commonly treated as stable traits that do not change in strength and can, thus, be measured by single-shot assessments (e.g., Schultheiss & Pang, 2007). If this were the case, the degree to which associated feature codes affect the selection of event files should not change over time, and the kind of impact on selection should depend on the agent’s need profile: individuals with a high need for achievement should show a stronger bias towards event files that generate achievement-relevant action effects than individuals with a low need for achievement, and so forth. And yet, the result would be a pseudo-explanation. The concept of a biological drive can be taken as an umbrella term for mechanisms taking care of the basic energetic resources of an organism. Even if the individual mechanisms may not yet be completely understood, it makes sense to consider that and how they might interact with feature codes that affect event-file selection. But the same does not hold for the concept of acquired needs. The concept does not stand for some definable mechanisms that we can investigate separately but serves as a placeholder for observed behavioral regularities. Hence, the concept of need for achievement, say, signifies the explanandum and can, thus, not serve as explanans (see Hommel, 2019a, 2020). If we, thus, assume that an individual with a high need for achievement has a strongly activated feature code that biases event-file selection towards actions that are more likely to generate achievement-related outcomes, we need to explain why the feature code is so strongly activated without referring to a hypothetical construct that merely summarizes, but does not yet explain, empirical observations that suggest a strongly activated code.

We are facing the exact same challenge when considering incidental goals. Various authors have pointed out that goals, once the agent is committed to them, are particularly sticky (Hollenbeck & Klein, 1987). Lewin (1936) suggested that committing oneself to a goal creates a kind of tension in one’s cognitive system that seeks for relaxation very much like a biological drive seeks for reduction (which among other things leads to a certain stickiness of goal-related representations until the goal is reached: Goschke & Kuhl, 1993; Zeigarnik, 1927). Given this strong similarity of goals to biological needs, Lewin considered goals as “quasi-needs” that work exactly the same way but differ in origin. Along the same lines, Klinger (2013) suggests that self-commitment turns mere motivation into goal-striving which, among other things, keeps the respective goal active until the intended outcome has been achieved. Commitment to the goal was also considered crucial to engage in actual goal-striving by Locke and colleagues (e.g., Locke et al., 1988) or Gollwitzer and Oettingen (2011). Indeed, there is massive evidence suggesting that self-reported commitment to the goal is the central predictor of successful performance, especially in difficult tasks (Hollenbeck & Klein, 1987; Klein et al., 1999)—but we lack a mechanistic understanding how commitment works, what it does, and how it manages to translate a goal that people just have into a goal that they actively pursue and that is as sticky as Lewin (1936) and Zeigarnik (1927) were suggesting.

As elaborated in more detail elsewhere (Hommel, 2021), the selection criteria associated with needs and goals one is committed to might become part of one’s self-representation (a kind of Me-file), so that they become a defining feature of an agent’s self-perception. There is evidence that people’s self-representation is rather malleable and likely to integrate feature codes that are perceived to systematically co-vary with features of oneself; e.g., an artificial hand or face is perceived as a part of one’s own body if it moves in synchrony with one’s own movements (e.g., Ma et al., 2016). This implies that selection criteria that an agent is repeatedly exposed to, like when growing up in a family that consistently emphasizes the importance of personal achievement (which in turn seems to be associated with strict feeding schedules and toilet training: McClelland & Pilon, 1983), or that is associated with otherwise acquired personal values, or that the agent is socially expected to heed, like in an experimental study, will become a long- or short-term ingredient of the agent’s Me-file. Given that each awake individual is constantly perceiving him- or herself, selection criteria that are integrated into the Me-file will, thus, be consistently primed and, thus, enjoy a consistently high level of activation—they, thus, become as sticky as selection criteria associated with biological drives.

## Having (almost) no goals

Self-help books often recommend being less ambitious and more open to new experiences in order to overcome everyday life stress and other troubling experiences. In particular, some popular meditation techniques explicitly aim at reducing cognitive control and self-regulation to improve mood and happiness. For instance, open-monitoring meditation (OMM) techniques encourage the meditator to become non-reactive and non-judgmental with respect to possible upcoming thoughts and emotions (Lippelt et al., 2014; Lutz et al., 2008). Brain-imaging studies have indeed revealed that this kind of meditation reduces functional connectivity related to intentional focusing and memory retrieval, and increases detachment from autobiographical memory (Fujino et al., 2018), suggesting that OMM reduces the impact of goals and undermines goal commitment. This fits with behavioral observations showing that OMM broadens the attentional scope and facilitates the integration of events over time (Slagter et al., 2007), facilitates dealing with unexpected events (Valentine & Sweet, 1999), and promotes divergent thinking and mental flexibility (Colzato et al., 2012). Techniques like OMM have been assumed to induce a metacontrol state that reduces the impact of goal-related criteria on the selection of event files and the degree to which alternative event files inhibit each other (Hommel & Colzato, 2017b). What does GOALIATH imply with respect to the idea to reduce stress and increase satisfaction by applying techniques that reduce the impact of goals on behavioral control?

One obvious implication would be that goals of different origins are likely to change their relative priority. Job and social requirements often promote a priority of short-term goals over more chronic goals like acquired needs or biological drives. This may bring several challenges with it: in particular, the short-term goals, often adopted from, or given by others, may conflict with the goal criteria associated with acquired needs and biological drives in prioritizing different kinds of actions. This is the psychological core idea underlying alienation (Marx, 1844/1964) and suspected to induce cognitive dissonance (Festinger, 1957), and a discrepancy between what has been called actual or real and ideal self in the literature (Higgins, 1987; Rogers, 1961)—with substantial risks for mental health (e.g., Heidrich, 1999). Reducing the impact of goal criteria, and especially those of the more fragile temporary goals, would, thus, be likely to relax possible tensions between incompatible short-term and long-term goals or goal criteria. Given that event-file selection emerges from competition between event files that match at least some of the goal criteria, reducing the impact of goal criteria would also imply less competition. As competition has been suspected to induce negative affective states (Botvinick, 2007), this would mean that applying fewer goal criteria may improve people’s mood.

Reducing the impact of goal criteria can furthermore be assumed to affect perception and creativity. According to TEC, perception works exactly like action in that both consist in selecting the event file that best characterizes (i.e., feature-overlaps with) the experienced or to-be-produced event. Reducing selection criteria would, thus, make both action selection and perception less selective and more variable, which among other things would result in a broader scope of perception. This would also allow for more exploration in mental or overt action and action control, as has been shown in creativity tasks (Colzato et al., 2012). Given the evidence that engaging in more divergent activities improves people’s mood (Akbari Chermahini & Hommel, 2012), this supports the expectation that reducing the impact of goal criteria improves people’s happiness and well-being. Hence, taken altogether, predictions from GOALIATH are consistent with at least some of the claims that have been made with respect to open-monitoring types of meditation.

## Further implications

The assumption that human goal-directed behavior emerges from the concerted impact of selection criteria that differ with respect to their internal support (by biological drives, acquired needs, self-related or other kinds of special files) has important implications for theorizing and experimenting about cognitive and action control. One implication concerns the way action goals are operationalized in most laboratory studies on control processes. For experimental reasons, these studies do not make any use of natural goals that participants may bring to the lab but ask for arbitrary and meaningless responses to arbitrary and meaningless stimuli. This means that the instructed goals do not have any support from, and thus conflict with existing goal criteria and existing supporters of these criteria, such as drives, needs, temporary intentions, and so forth, which in turn raises two obvious problems: the instructed goal will be likely to conflict with other ongoing goals and it will be rather difficult to maintain over time. If so, it is unsurprising that theories of cognitive control place a lot of emphasis on goal maintenance, the inhibition of unwanted responses, and the switch to uncommon tasks (e.g., Logan, 1985; Miller & Cohen, 2001; Monsell, 1996). However, my present considerations suggest that these functions, and thus the mechanisms underlying them, might not be representative for everyday goal-directed behavior. They may rather reflect people’s ability to deal with arbitrary, artificial, and personally meaningless tasks—an important feat that will, however, not be of much use outside the lab. Hence, our theories of cognitive control may cover no more than just a small part of the goal-directed behavior people perform.

The second implication is that (presumably encouraged by the Rubicon logic) existing theorizing systematically underestimates the existence of other goals than the instructed one. The widespread believe in the Rubicon logic, according to which other goals may play a role for pre-decisional but not for post-decisional processes, has justified this ignorance. However, given the increasing evidence that goals keep affecting action control even after decisions have been made, acknowledging the impact of multiple goals will lead to fundamental changes in the interpretation of performance characteristics. For instance, once researchers admit that curiosity and novelty processing cannot simply be switched off, the processing of novel, curiosity-satisfying stimuli can no longer be interpreted as a breakdown of attentional control. This undermines the common interpretation of corresponding effects like the Stroop effect, flanker effects, and oddball effects, as well as the theories that rely on such interpretations.

A third implication relates to dual-route theorizing about action control (e.g., Verbruggen et al., 2014). Almost all existing control models have incorporated the historical distinction between will and habit (or some of the newer disguises like intentional/automatic; model-based/model-free processing), which suggests a continuous battle between truly intentional, endogenously activated, willed behavior on the one hand and stimulus-driven, exogenously triggered, involuntary action tendencies on the other (Hommel, 2019b). As argued elsewhere, none of these distinctions survives critical conceptual analysis and they do not do a good job in organizing empirical findings either (Hommel & Wiers, 2017; Hommel, 2019c). GOALIATH adds to this skeptical view by suggesting that most real-world actions would count as non-intentional according to the will-habit perspective. Ironically, the more a goal would reflect the agent’s ongoing interests and concerns, the less this goal would rely on the cognitive-control machinery that dual-route models claim to underlie truly intentional action, and the more effortless and automatic this impact would unfold. This implies that dual-route theorizing considers actions to be truly intentional to the degree that they do *not* reflect people’s real interests and wishes—which undermines its ambition to model human action control. In contrast, GOALIATH suggests that both routes of dual-route theorizing work exactly the same way, except that some of the operative selection criteria are consistent with the expectations and interests of the outside observer (like the psychological experimenter) while others are not. This attempt to account for both so-called voluntary and so-called involuntary movements by means of the same mechanism might be considered a renaissance of what Stock and Stock (2004) have coined the British root of ideomotor theory: Taking up considerations of Laycock (1845), Carpenter (1852) suggested that many apparently pathological spasms and reflex-like motor tics observed in patients might be produced by the exact same ideomotor mechanism that generates “normal” actions, only that the ideas driving this mechanism might be overly context dependent or inappropriate. In other words, all behavior might be intentional, but some intentions may be less functional than others.

﻿A fourth implication is more meta-theoretical in nature. In addition to phenomenon-specific models and hypotheses, a number of grand theories have been proposed to account for large portions of human behavior. While these accounts undoubtedly hold greater promise than piecemeal theorizing, they in many cases are built on one single principle that is then used to define optimal choices and behavior: maximizing reward in economic approaches or maximal predictability of one’s environment in predictive-coding approaches. If the idea underlying GOALIATH, that decision-making and action control emerge from the interplay of unrelated contributors, is correct, single principles and optimality ideas based thereupon are unlikely to provide exhaustive accounts of human behavior. This is not to say that they do not capture important aspects of human action control: it may very well be that they characterize one of the contributors, and the better they are supported by empirical evidence the more likely this is the case. But it would be hard to see how and why a single principle and, thus, a single criterion would represent all the different drives, needs, and preferences that people bring to the real-world. It would also be hard to see how Darwinian evolution would have generated such a guiding principle in the first place. In contrast to common believe, and common argumentation to motivate single-principle approaches, Darwinian evolution is unlikely to optimize a particular species. If it would, the Darwinian mechanism would lose its key strength, namely, a considerable variability among the members of a species, which allows adaptation to even dramatic changes in the environment. What Darwinian evolution does, is merely to eliminate the least fitting members or species—it, thus, shapes through elimination but not optimization.

A fifth and final implication is also meta-theoretical in nature. Consider why both laypersons and researchers find dual-route theorizing so convincing. The reasoning underlying this logic has a long-standing history. For instance, Plato’s theory of soul (an obvious pre-runner of the Freudian tripartition of superego, ego, and id) already distinguished between three components, namely, reason (logos), emotion (thymos), and desire (eros; Jones, 2009), which Plato likened to a charioteer (reason) trying to control two horses pulling into different directions. However, given that using different concepts to describe people’s behavior from different perspectives does not imply that the underlying mechanisms are different or non-overlapping (Hommel, 2019a)—the charioteer and the pulling horses may turn out to be the same animal. And yet, the tendency of psychological theorizing to take the existence of different concepts to imply different underlying systems or mechanisms has fueled the idea of reason, which is commonly translated into conscious reasoning, is constantly challenged by counteracting forces that reason has to withstand to do the right thing. Apart from the obvious religious undertones of this figure of thought, it also carries a scientifically questionable ideology that suggests that for actions to count as truly intentional, the agent has to overcome personal needs and interests by brute intellectual force. It is this predominance of rational thinking over other personal needs and interests that according to Weber (1905/2002) is characteristic of the “spirit of capitalism” and its deeper roots in the Protestant working ethic, and it is this predominance that the concept of alienation refers to (Marx, 1844/1964). GOALIATH provides a less ideological platform to empirically investigate possible effects of culture, ideology, economic systems, and educational styles on the way people integrate contributions of different sources to action control.

## Conclusions

It was my ambition to develop a mechanistic theory of the underpinnings of human goal-directed behavior. As I have pointed out, goals are first and foremost concepts in the heads of observers that should be not uncritically attributed to the agents whose behavior we are trying to understand as layperson or researcher. Then I have rejected the most radical pessimistic stance that considers goal-directed behavior as a purely emerging property of the hardware of the agent, in interaction with the current circumstances. Hence, I argue that people do have internal states that are actually guiding their behavior, which allows them to predict the outcomes of their actions beforehand, to immediately correct their actions if something went wrong, and to act differently under identical stimulus conditions. I further argue that this behavioral guidance is provided by feature codes serving as selection criteria. These criteria create competition between event files that contain action-effect codes that are matching one or more of the criteria, and this competition eventually settles into a solution that favors the best-matching event file, at least under ideal circumstances. The criteria are associated with, and in a sense representative of various sources, including biological drives, acquired needs, and short-term, perhaps even arbitrary, instructed aims. Selection will, thus, always be a compromise that tries to satisfy various criteria related to different driving forces, which are also likely to vary in strength over time. Hence, even if one chooses to call the total of currently active selection criteria “goal”, this “goal” will not likely consist of a coherent structure or process but rather a patchwork of goal components that may be contradictory or in conflict with each other. What looks like goal-directed action, thus, seems to emerge from, and represent an attempt to satisfy multiple constraints with different origins, purposes, operational characteristics, and timescales—which among other things does not guarantee a high degree of coherence or rationality of the eventual outcome. As a consequence, it is indeed unlikely that we will be able to locate individual goals in particular functional or neural locations (see Uithol et al., 2014), but studying the interplay between selection criteria and event-file activities seems a challenge we might be able to meet.

## Footnotes

1 These three criteria can indeed be considered my minimalist working definition of goals: a goal is whatever allows an agent to (1) represent aspects of the outcome of his/her action beforehand; (2) be sensitive to the degree to which expected and actual outcomes differ; and (3) behave differently in the same situational context.

2 It may be interesting to note that this dynamic view on the relationship between perception and action is not too different from the cybernetic approach of Powers (1973). However, while this approach would have the potential of forming the basis for an integrated perception–action system like TEC, it has been interpreted in a rather asymmetric fashion by emphasizing the perceptual aspect and downgrading action to a function that only serves the purpose of compensating for disturbances. Such an asymmetry is inconsistent with evolutionary considerations that TEC sympathizes with, as what counts in Darwinian evolution is doing the right thing but not perceiving the right thing. A similar inconsistency exists between TEC and predictive-coding approaches (Friston, 2012; Parr & Friston, 2017), as the latter also consider action as a mere hypothesis-testing tool to serve the more important purpose of holding the right views on the world.

3 Note that the event-file concept has been applied to both temporary short-term bindings, as in studies of trial-to-trial effects (e.g., Hommel, 1998), and to long-term feature bindings, as in the context of ideomotor learning. Even though it makes sense to assume that the structure of short-term and long-term bindings are comparable, it is not yet entirely clear how they relate to each other (Colzato, Raffone & Hommel, 2006; Hommel & Colzato, 2009). It might be that short-term bindings are simply amassed, similar to Logan’s (1988) instances, so that a long-term binding consists of the total (and, hence, the functional average) of all retrieved short-term bindings. It might also be that individual short-term bindings are forgotten sooner or later but the basic combinations of features that are shared by many short-term bindings are extracted and kept as a kind of generic event file (Kachergis, de Kleijn & Hommel, 2015)—so that the relationship between short-term and long-term bindings would be similar to the distinction between episodic and semantic memories. While further research is necessary to disentangle these and other theoretical options, the particular outcome is not relevant for present purposes, however.

4 Gollwitzer (1990) does admit that this metaphor should not be taken too seriously, as some degree of overlap between pre-decisional and post-decisional phases may be possible. Nevertheless, it may be the case that the metaphor turns out to be more misleading than useful.

5 Note that everything that applies to the concept of goal (that it exists in the head of the observer but does not necessarily correspond to clearly defined, separable systems or mechanisms in the head of the observed) also applies to the concept of needs, so that every attempt to conceive of an exhaustive list of all needs seems futile and theoretically misled.
